# Reflections on integrated care from those working in and leading integrated respiratory teams*

**DOI:** 10.1080/17571472.2017.1421020

**Published:** 2018-01-01

**Authors:** Nicola J. Roberts, Mike Ward, Irem Patel, Janelle Yorke, Martyn R. Partridge

**Affiliations:** a Institute of Applied Health Research, Glasgow Caledonian University, Glasgow, UK; b Sherwood Forest Hospitals, Co-chair IMPRESS, NHS Midlands Respiratory Lead, Mansfield, UK; c Integrated Care, Kings College Hospital London, London, UK; d School of Nursing, Midwifery and Social Work, University of Manchester, Manchester, UK; e Respiratory Medicine, NHLI, Imperial College London, London, UK

**Keywords:** Integrated care, consultant role, respiratory

## Abstract

The concept of integrated care has been advocated for many years to address some of the challenges faced by the NHS. This report examines the experiences of respiratory healthcare specialists working in an integrated role. Twelve qualitative telephone interviews were undertaken with a range of integrated respiratory specialists and their teams working in both hospitals and the community. A descriptive and thematic approach to data analysis was adopted.

Participants were very enthusiastic about their roles and saw themselves as ambassadors for this new way of working. Several key themes were identified from the analysis which participants identified as barriers or enablers to the successful undertaking of an integrated respiratory specialist role. These included the participants’ previous work experience and background, the range of multi-disciplinary expertise within or needed for the team, the structure of the team leadership and the measurement of outcomes to evaluate the team. Participants identified the need for clear job descriptions and roles, shared training and standards and appropriate outcome evaluation. More research is needed to understand how these new ways of working are developing and how they can be evaluated.

**Why this matters to me**

As people live longer, with increasingly complex health issues there is an ever increasing pressure on the NHS. As a result of this we need to change and improve the way we deliver healthcare and one way to do this is by integrating care between different healthcare settings, and different healthcare professionals changing the way that we work, and by changing some of our roles. This study looks at how new integrated respiratory specialists can help streamline and join up services between health settings and the barriers and enablers to doing this.

**Key message**

Integrated respiratory specialists working across primary and secondary care settings may help to provide better coordinated services for patients but there is a need to systematically evaluate these emerging roles.

## Introduction

The performance of the United Kingdom (UK) National Health Service (NHS) continues to be the focus of much attention and it is clear that it cannot continue to deliver care in the same way. This is in part due to an aging population with increasingly complex healthcare needs [1] such as those with one or more long term condition [2]. A decade ago the British Thoracic Society produced a discussion paper on integrated care, introducing the concept of the integrated respiratory specialist; a specialist who might work both in hospital and in the community [3].

Integrated care is defined by the World Health Organization as ‘The management and delivery of health services so that clients receive a continuum of preventive and curative services, according to their needs over time and across different levels of the health system.’ [4]. Integrated care takes many different forms, it can focus on all or some sectors of health care (i.e. primary, secondary, social). The integration can be virtual or involve fulling merging services pooling budgets and at different levels (macro, meso, micro).

Integrated care is now a key priority according to national policies and publications such as the ‘Five year forward view’ and the ‘Future hospital: caring for medical patients’ reports from the NHS and Royal College of Physicians [5–7]. A recent report on health and social care integration has shown that there is no ‘compelling evidence’ to suggest that ‘integration in England leads to sustainable financial savings or reduced hospital activity’ [8]. However, it is important that this process is ‘led, managed, and nurtured over time’ [9]. Working in an integrated way will involve moving from the traditional hierarchical organisation of care based on clinical disciplines towards delivering patient-centred care based across sectors. This will take the specialism nearer the patient but some may fear that working in the community leads to a loss of specialist hospital skills.

Partridge and Baxter [7] have identified some of the activities that integrated respiratory specialists are reporting that they undertake as part of an integrated specialist role. These included providing medical input into multidisciplinary team (MDT) reviews, early supported discharge or admission avoidance schemes, pulmonary rehabilitation, smoking cessation services, community nursing teams and community clinics. Other activities also include home visits and virtual GP clinics, as well as educational activities for general practitioners and practice nurses.

This report examines the views and experiences of respiratory healthcare specialists involved and leading integrated respiratory teams who are already undertaking this specialist role.

## Methods

Care for those with respiratory illness has traditionally been provided both in the community and within hospitals. Bridging those two types of care has happened for the care of those with tuberculosis for decades. More recently home oxygen services and assisted discharge schemes for those with exacerbations of chronic obstructive disease have been described and evaluated with beneficial results. Those providing such care have always come from several health care professional backgrounds but more recently a greater degree of integration of services for those with respiratory conditions has developed involving multiple professionals working according to agreed protocols to provide specialist respiratory care of the same standard to patients whether at home or in hospital. Such a change to a less hospital centric approach involves sessional commitments to community respiratory care by respiratory physicians, nurses, general practitioners, physiotherapists and a variety of other health professionals and for those previously trained in hospital based work this necessitates new structured training within the community. This report explores the experiences of those working in this new way.

The steering group consisted of individuals with a range of roles (clinical academic, consultant physician, integrated respiratory specialist, respiratory nurse educator, health services researcher) all with an interest in integrated respiratory care. Utilising the knowledge and expertise of the group and British Thoracic Society resources opportunistic sampling was used to identify potential participants working in an integrated respiratory specialist role across the UK from knowledge of personal networks, BTS networks and other reliable sources. A qualitative approach using semi-structured interviews was adopted. 16 individuals were contacted via email and 12 agreed to participate (12/16, 75% response rate). Participants were all working either as a lead or co-lead of an integrated respiratory team or as a key team member (Specialist Trainee (ST), General Practitioner (GP), or pharmacist). Potential participants received information about the study purpose (participant information sheet) and contacted the project lead to opt-in to the study.

Telephone interviews were undertaken (NJR) and the interviews lasted approximately 70 min using an interview schedule to elicit views around several topic areas including but not limited to: the creation of integrated posts, details of job roles and activities, and barriers and enablers about this new way of working. All of the interviews were audio-recorded and transcribed verbatim with any identifiable data removed. Written and/or verbal consent was sought for all participants. A descriptive and thematic approach to data analysis was adopted, raw data were read and themed by one researcher, then validated by an additional researcher [10]. Quotes used from specific interviews are labelled with the interviewee number e.g. (I-1). All of the study materials were reviewed and refined within the project Steering Group and ethical approval was obtained from a University ethics committee (SHLS) (HLS12/119).

## Results

Twelve qualitative interviews were conducted with a selection of integrated respiratory specialists which included six consultant physicians, two nurse consultants, one physiotherapist consultant, one speciality trainee (ST), a general practitioner (GP) and a pharmacist. Some were in newly created full-time specialist posts and others had re-developed their posts to include integrated sessions in the community. Of the nine integrated specialists interviewed (5 Female; 4 Male), three were funded through secondary care, two were funded through the community and four were joint funded. Details about the integrated services in each team are shown in Table 1.

**Table 1.** Components of an integrated respiratory service.

Multidisciplinary team meetings	Weekly in patient (*n* = 2)
	Yes (*n* = 7)
Community clinics	Yes (*n* = 4)
	Hospital based (*n* = 1)
	No (*n* = 2)
	Not at the moment (*n* = 2)
Virtual clinics	Email service (*n* = 1)
	Yes (*n* = 2)
	MDT meeting (*n* = 1)
	Virtual ward round (*n* = 1)
	No (*n* = 4)
Education for community staff	Yes (*n* = 9)
Domiciliary visits	Yes (*n* = 9)
Producing clinical guidance for avoidance/other pathways	Yes (*n* = 9)
Supporting quality assured spirometry	Trained (*n* = 1)
	Provide training and education (*n* = 7)
	Yes within rehab (*n* = 1)
Respiratory reviews in acute medical unit	Yes (*n* = 6)
	No (*n* = 2)
	Covered by colleagues (*n* = 1)
Oxygen assessment service	Yes (*n* = 6)
	On caseload only (*n* = 1)
	Nurse run service (*n* = 1)
	For COPD patients only (*n* = 1)
Smoking cessation service	Yes (*n* = 4)
	Referrals (to hospital service, or smoking cessation service, *n* = 3)
	Input from charity in clinics (*n* = 1)
	Linked to local authority services (*n* = 1)
Advance care planning	Yes (*n* = 7)
	Part of a clinic (*n* = 1)
	Yes (joined by community palliative care team for MDT (*n* = 1)
Other roles	Clinical ethics committee, drugs and therapeutics committee (*n* = 1)
	Inpatient ward round for inpatients – 3 times a week (*n* = 1)
	Care planning conference, (plus mental health, psychiatrist, social worker, medical team OT and patient plus family + plus GP) (*n* = 1)

Several barriers and enablers to delivering integrated care were identified from the interviews; these included the participants’ prior experience and background, the team expertise, the presence of a second co-lead as part of the team leadership and need for appropriate outcomes assessment of the integrated team (Table 2).

**Table 2.** Barriers and enablers to delivering integrated care.

*Participants’ background*	Enabler	‘Training flexibly means you’re working in slightly different ways to most of your peers, so you have to negotiate your role sometimes you have to be quite thick skinned, you need to be clear of what the value of your role is when it’s slightly different to the traditional model and that’s actually what I’ve had to continue to do as a consultant, so, looking back, that was also quite helpful.’ (I-2)
	Enabler	‘One of the key things about doing flexible training and flexible working is you have to be able to have time to reflect and think and I think that has been a really big thing in terms of being able to innovate.’ (I-8)
*Expertise within an integrated team*	Enabler	*General practitioners* – ‘What a GP can bring to an integrated team is … the consulting skills, dealing with uncertainty and I think that the GP who is aware of the politics of their local borough or whatever, is kind of vital.’ (I-10)
	Enabler	*Pharmacists* – ‘they [pharmacists] were so high value and I think they are absolutely an integral part of the team’ (I-10)
	Enabler	*Flexible trainee post* – ‘I don’t think you can understand an integrated service unless you know where the GPs are coming from and being able to form personal relationships with those people is really important ‘ (I-4)
	Enabler	*Flexible trainee post* – ‘It would be a routine part of SPR’s training for all of them…I think for anyone who’re going to be a general respiratory physician, it should be part of their normal training that they come through an integrated service,’ (I-2)
	Enabler	*Flexible trainee post* – ‘it’s been the best job I’ve done on my rotation’. (I-4)
*Training*	Barrier	‘And I think we should all join that up so that it might be that there isn’t one in every hospital but there’s one in every region and that we then make sure that registrars are freed up regionally to come through these services and that happens for other things like cancer trials to CF. If you haven’t got that going on in your hospital, each year you have your review of training, you make sure you spend a couple of weeks even or a couple of months even as an observer.’ (I-2)
	Barrier	‘It need support through the professional specialist organisations like BTS… also they need government buying.’ (I-11)
	Barrier	‘Also needs support from within acute trusts and CCGs’ (I-11)
	Barrier	‘I don’t think people see it as a potential speciality’ (I-1)
*Team leadership*	Enabler	‘… I’d have loved to have a nurse consultant or somebody supporting the community role side of things, so I think a collaborative role with two people is much better than one person trying to straddle lots of different bits of it. I think a physician in crucial to this – there has to be a physician somewhere along the line’ (I-2)
	Barrier	‘I think depending on how many sessions of your clinical time you spend doing this it can deskill you because the more you spend the less time you spend in the hospital and I think a truly integrated clinician is one that will do both’ (I-7)
	Barrier	‘[I] don’t line manage any of them because as a nurse consultant’
	Barrier	‘Both of the managerial teams [acute/community] want a bit of me, and want me to be their lackey rather than the other person’s lackey’( I-9)
*Evaluating the value and outcomes of the integrated team*	Barrier	‘Community clinics that we ran were loved by the patients so patient satisfaction was really, really high but it was again it was coming down to cost and I don’t think, we couldn’t demonstrate cost effectiveness within the short time that we had.’ (I-12)
	Barrier	‘The idea that you might look at the cost of the given patient’s care across different providers and somehow make sure whatever rewards come from saving an admission is shared across all providers all of that is extremely challenging and hasn’t been thought through and that’s what really needs to be in place for these things to work properly otherwise you’re constantly coming up against barriers to do with how things are organised’ (I-2)
	Barrier	‘The management which has to be aligned along the lines of patient’s quality of care rather than financial incentives.’ (I-9)
	Barrier	‘We need to do is to prove that these roles are valuable first and there isn’t enough valuation yet to warrant although the commissioners, the other way of doing it is to get the commissioners to believe that this is the way forward because at the end of the day if the commissioners want it somebody has to provide it.’ (I-9)

### Participants’ background

Participant’s training and experiences were seen as an enabler to undertaking an integrated post. Several of the physician participants interviewed (3/9, 33%) had previously completed flexible training which permitted participants to have experienced integrated care. Participants commented that this had helped with their integrated roles, and may have made their specialist consultant post more accessible. One consultant physiotherapist had previous experience working in an extended scope physiotherapy role. These different experiences may have helped individuals be more aware of integrated care as a career and they may have more relevant experience for an integrated role.

### Expertise within an integrated team

The teams varied in size, types of staff and location dependant on the demands of the funder and service population (e.g. 24/7 cover). In some cases there were multiple teams with some hospital based, and some community based however there were some which were completely contained in the community alone.

All had a mixture of nursing and physiotherapy staff, assistant practitioners (e.g. physiotherapy and/or occupational therapy assistants for pulmonary rehabilitation), healthcare assistants (HCAs) and rehabilitation technicians. Physician integrated specialists often rotated with other respiratory specialists to cover acute medical units (AMU) and wards. For most teams, specialist services were sought as needed for example, palliative care specialists and clinical psychologists. In some teams other specialist expertise has been added to the team structure including occupational therapists, smoking cessation advisors, dedicated integrated GP time and community pharmacists (involved in MDT role). Additional resources suggested by participants included having the following staff as part of their team; social workers, occupational therapists, psychologist, nutritionists and smoking cessation advisors. Four of the teams reported having psychotherapists, psychologists or clinical psychologists as part of their teams. Two teams had smoking cessation expertise within the team and at least one team would like to have this expertise within the team. Only two teams included dedicated GP (*n* = 1) and pharmacist time (*n* = 1). Although teams worked closely with GPs, their potential role within the team was considered an important asset. Pharmacists were also suggested to potentially play an important part of the integrated respiratory team, although they were not routinely part of the MDT.

One key barrier to delivering integrated care included line-management issues either for the consultant roles who often had several line managers. In addition often the integrated respiratory specialist did not line-manage their teams.

Efforts have been made to introduce junior medical staff and other healthcare professionals to this new way of working by highlighting the integrated team roles. For example a new supernumerary specialist registrar trainee post in integrated respiratory care was designed specifically to provide training in integrated care including components such as virtual ward rounds, virtual clinics, home visits, delivering GP respiratory education and specialist advice, and delivering training to HCPs. Overall the participant who was currently in the trainee post had positive experiences, and other participants felt it was important that trainees and junior staff are exposed to this new way of working as a potential career path. This new way of working as a potential career path – is currently not well promoted, with one participant stating ‘I don’t think people see it as a potential speciality’ (I-1).

### Team leadership

Five participants were leads of integrated teams which had two joint leads. Specialists working as sole leads stated that there was value in having an additional specialist to complement their skills and allow collaboration. The role of a physician as part of the team was also felt to be important from a medical and clinical responsibility perspective. Some teams had some consultant support, however time input varied. It was suggested that allocated sessions in the community and in hospital for specialists would ensure adequate liaison with colleagues in both spheres and address issues around isolation and de-skilling.

### Evaluating the value and outcomes of the integrated team

As part of the rolling out of any new service, evaluation and research is needed for commissioners and other stakeholders. Participants indicated that the service and its components (e.g. pulmonary rehabilitation, community clinics, primary care registers reviews) should be evaluated to ensure it is fit for purpose and should be evaluated using appropriate measures.

A range of outcome measures were used by the participants’ teams to evaluate the team. These often included patient, carer and team member satisfaction surveys or used existing patient reported outcomes tools. However commissioners often focussed on other, more difficult to influence outcomes such as number of re-admissions or length of stay. It is also difficult to evaluate the value of a team and its members given the shared contribution, although there is an urgent need to show how valuable these roles are.

## Discussion and conclusion

Participants were very enthusiastic about their roles and saw themselves as ambassadors for this new way of working despite reporting that they often felt isolated and without recognition of the value of their new roles. Several of the participants in this study had undertaken flexible training, which in the past has been perceived as an ‘add-on’ often with sub-optimal clinical experience [11]. As part of this training, participants had a more hands-on role organising their training leading to different experiences that traditional training would not provide. Supernumerary training posts are much rarer now due to increased requests without a significant rise in funding. This highlights that new training and career opportunities are needed to promote and standardise this new pathway much earlier so that trainees and students can be exposed to this way of working.

The make-up and structure of the multi-disciplinary integrated team is very important. The presence of a multi- disciplinary team delivering evidence based long term care has been shown to be associated with more complete service delivery [12,13]. Participants discussed pulling together of a wide range of different health professionals as part of an integrated team. Although there were some issues around lack of shared training and the difficulty of managing or not managing other clinical specialities. The provision of two joint leads has been shown in this study to work well, with the importance of having a physician involved has also been emphasised. Leadership within integrated care is different, with a focus on outward thinking, innovation and collaboration across boundaries. Evidence suggests that with this type of knowledge sharing and collaboration this brings deeper understanding and insight [14]. Often careers outside of hospital medicine are seen as lacking, perceived as being less specialist. Raising awareness of this new way of working, and providing job specifications on how allocated sessions can be undertaken can help to overcome the views around ‘deskilling’ [15].

Table 1 showed the wide range of activities that some specialists were undertaking as part of their role, and it is evident that there is significant variation. Healthcare professionals need clear job descriptions and roles, including generalists and specialists to be able to work together within MDTs [9], and we need to look at this across specialities at who will be involved in delivering care, looking at shared roles and joint training [16]. This includes the provision of generic job descriptions for integrated respiratory consultant roles [17]. There needs to be some consistency and equity across the UK for these roles.

One of the participants in this study was a speciality trainee who reported positive experiences with this pilot post. As part of the evaluation views were sought from a training day and a significant number, 70% of registrars expressed an interest in integrated posts [18]. Planning needs explore how we get trainees involved and knowledgeable about these new career options using training curriculums, Deaneries and BTS to promote training opportunities [8]. Recent highlighting of new integrated roles will help for example, BTS trainee advisory group sub-speciality spotlight [19] and training courses on integrated care [20].

Many of the teams had access to other healthcare professionals outside of their team which can have limitations, for example, dieticians, smoking cessation experts, pharmacists and GPs. Dependant on how accessible these services are and the demand, careful consideration should be given to the benefits/costs of integrating this expertise within the team. Some components of the integrated respiratory team approach has been well evaluated (e.g. pulmonary rehabilitation, early supported discharge, admission avoidance schemes), in particular medication reviews [21,22]. Davies (2016) showed reductions in spend on inappropriate inhaled therapies for COPD in CCGs with integrated team. Pharmacists may have played or have the potential to play a crucial role in helping with medication management [23]. GPs, pharmacists and psychologists are often overlooked in their role in an integrated care team, several studies have highlighted the key role these team members can play in enhancing care, and reducing costs [9,24–26]. However this type of team role is often underfunded [17,27].

### Evaluating the value and outcomes of the integrated team

Participants raised the difficulties of evaluating the new integrated services balancing the wants and needs of commissioners and managers, as well as time restraints. However it is important that any new way of working is evaluated, and that effectiveness is measured. The use of specialist skills in the community (nurse or consultant) can help with improving diagnosis and treatment and reducing readmissions. The RCP’s Future Hospital project suggests a new role for hospital without walls, where there are no physical and mental barriers to delivering care anywhere without being limited to within the hospital walls. This utilises a population approach promoting joined up working across sectors to improve value i.e. outcomes and reduced costs [28,29]. Specialists and generalists need to work together to improve the health of the population. It is important that we ‘Measure what matters’ [16] measurement needs to focus on the experiences of patients and their outcomes from their care. Commissioning organisations are still financially motivated so their focus is on cost reduction/ cost-effectiveness or at least using funds wisely (i.e. value-based healthcare). The role of ‘commissioning for outcome’ negotiating with commissioners about outcome measures is an important issue [16].

Patient reported outcomes (PROMs) are a valuable tool to measure and improve the quality and outputs of the health service. The Darzi report recommended that they should play a bigger role [30]. A review of patient reported outcome measures for COPD in 2009 identified some useful generic PROMs such as SF-36, EQ-5D, COOP and HUI as well as COPD-specific instruments including CRQ, CCQ and SQRQ [31]. However administering these tools in reality in real-life practice can be time-consuming and often not well completed.

It is important that appropriate outcomes are agreed early on between providers and commissioners and that ongoing data collection and evaluation takes place in order to facilitate team learning and allow an assessment of the cost-effectiveness of such new approaches to care.

## Funding

This study was funded by the British Thoracic Society

## Disclosure statement

No conflicts of interest from any of the authors

## Authors contribution

All authors were involved in the research project including analysis and writing up the report and publications.
